# Analysis of Virulence and Antimicrobial Resistance Gene Carriage in Staphylococcus aureus Infections in Equids Using Whole-Genome Sequencing

**DOI:** 10.1128/mSphere.00196-20

**Published:** 2021-08-04

**Authors:** Sara V. Little, Andrew E. Hillhouse, Sara D. Lawhon, Laura K. Bryan

**Affiliations:** a Department of Veterinary Pathobiology, College of Veterinary Medicine & Biomedical Sciences, Texas A&M University, College Station, Texas, USA; b Texas A&M Institute for Genome Sciences and Society, College of Veterinary Medicine & Biomedical Sciences, Texas A&M University, College Station, Texas, USA; University of Nebraska Medical Center

**Keywords:** *Staphylococcus aureus*, antibiotic resistance, enterotoxins, genome analysis, horse

## Abstract

While Staphylococcus aureus is associated with significant morbidity and mortality in equids (horses, donkeys, and mules), few studies have performed whole-genome sequencing to fully categorize large collections of equine isolates. Such sequencing allows for a comprehensive analysis of the genetic lineage and relationships of isolates, as well as the virulence genes present in each, which can be important for understanding the epidemiology of strains and their range of infections. Seventy-two clinical Staphylococcus aureus isolates from equids were collected at the Texas A&M University Veterinary Medical Teaching Hospital between 2007 and 2017. Whole-genome sequencing was performed to characterize the isolates according to sequence typing, biofilm association, antimicrobial resistance, and toxin gene carriage. Of the 72 isolates, 19% were methicillin resistant, of which the majority belonged to clonal complex 8. Eighteen distinct sequence types (STs) were represented, with the most common being ST1, ST133, ST8, and ST97. Most isolates had weak or negative overall biofilm production. Toxin and antimicrobial resistance gene carriage was varied; of note, this study revealed that a large proportion of North American equine isolates carry the leucocidin PQ toxin (66% of isolates). One isolate (17-021) carried genes imparting lincosamide and high-level mupirocin resistance, a combination not previously reported in equine-derived S. aureus isolates.

**IMPORTANCE** This is one of the first studies to perform whole-genome sequencing (WGS) of a large collection of Staphylococcus aureus isolates, both methicillin resistant and susceptible, collected from horses. A large proportion of the isolates carry leucocidin PQ (LukPQ), making this one of the first reports of such carriage in the United States. The presence of lincosamide and high-level mupirocin resistance in a methicillin-susceptible S. aureus (MSSA) isolate highlights the importance of MSSA as a reservoir of important antimicrobial resistance genes. As microbial resistance genes on mobile genetic elements can pass between S. aureus strains and livestock-associated strains can be transferred to humans, these findings have important public health implications.

## INTRODUCTION

Methicillin-susceptible and -resistant strains of Staphylococcus aureus (MSSA and MRSA, respectively) are associated with significant morbidity and mortality in horses (1). The prevalence of S. aureus colonization in horses on farms ranges from 1% to 8% in Canada (2) and 4% to 39% in Europe (3–5). Hospitalized horses reportedly have a colonization prevalence of 40% to 50% (4, 6, 7). The prevalence of MRSA in healthy horses ranges from 0% to 6%, with most studies reporting a 4% to 5% prevalence (1, 2, 8–14). Horses are most frequently colonized and infected by staphylococci on the skin, respiratory tract, and genital tract (7). S. aureus can be grouped based on multilocus sequence type (MLST), *spa* type, and ribosomal MLST (rMLST) with broad groupings organized into clonal complexes (CCs) (15–17). MRSA can also be further divided into *dru* and SCC*mec* types based on differences in the methicillin resistance cassette (18, 19). MRSA has important public health implications because it can be transferred between humans and horses, as well as among groups of horses (20, 21).

Antimicrobial resistance, often to multiple classes of drugs, is a significant problem in S. aureus, and resistance genes are frequently carried on mobile genetic elements (MGEs) and can be passed among isolates. Trimethoprim-sulfamethoxazole (TMS) drugs and aminoglycosides are commonly used to treat infections in horses as they provide broad spectrum antimicrobial coverage (22, 23). The genes conferring high-level aminoglycoside resistance in S. aureus are *aac (6′)-Ie/aph (2′')* and *aph (3′)-IIIa*, and kanamycin-neomycin resistance genes *ant (4′)-Ia* or *aadD* are carried on plasmids (24). The major chloramphenicol resistance gene *cat* is carried on the plasmids pC221 and pC223 (25), while TMS drug resistance is mediated by the *dfr* genes (26). In horses, long-term treatment of staphylococcal eye infections with topical fluoroquinolones is a risk factor for developing resistance to fluroquinolones and other drug classes (27). The *tet* genes encode efflux pumps mediating tetracycline resistance, while the related multidrug efflux pump encoded by *norA* provides fluoroquinolone resistance (26). In France, the proportion of equine S. aureus isolates resistant to aminoglycosides, tetracyclines, or sulfonamides has increased from 2016 to 2019, with multidrug-resistant (MDR) strains rising from 26% to 52% (28).

Macrolides such as erythromycin are not commonly used to treat staphylococcal infections, but their widespread use in treating other equine bacterial infections, such as pneumonia attributable to *Rhodococcus* spp., has provided secondary exposure to S. aureus, resulting in resistance genes (*erm*, *msrA*, and *mph*) becoming more common (23, 26). Treatment with lincosamides, such as clindamycin, is typically contraindicated in horses due to the potentially fatal enterocolitis complications that arise from their use, but resistance (due to the lincosamide nucleotidyltransferase *lnu* genes) is occasionally reported in animal-derived staphylococci and lincosamides are used to treat soft tissue infections in people (29). Mutations of the gene encoding the β subunit of the bacterial RNA polymerase *rpoB* in MRSA strains can confer rifampin resistance (26, 30). Fosfomycin is used to treat MDR strains, but resistance mediated by *fosB* is an emerging problem and is often observed in equine-derived isolates belonging to CC8 (31, 32). Carriage of the purported quaternary ammonium compound resistance genes *qacA/B*, *qacC*, or *qacJ* is also relatively common in S. aureus isolates, particularly of the ST1 lineage (33, 34), while high-level mupirocin resistance is mediated by transfer of the plasmid carrying *mupA* (26). Mupirocin is sometimes used to decolonize the nasal and pharyngeal passages of MRSA-positive people, but its use in equids is limited to topical treatment of pastern folliculitis that is resistant to TMS (26, 35).

Toxin genes in staphylococci are often carried on large MGEs known as pathogenicity islands that can be horizontally transferred. In S. aureus, pore-forming toxins include alpha-hemolysin (Hla), leucocidins, and phenol-soluble modulins (36). Important S. aureus leucocidins include Panton-Valentine leucocidin (*lukSF-PVL*) and leucocidin PQ (*lukPQ*). The purported equine host-adapted leucocidin is *lukPQ* and causes death of neutrophils, while PVL is a significant virulence factor in human-associated strains (37). Staphylococcal superantigens cause indiscriminate stimulation of T lymphocytes resulting in a mass release of cytokines that can cause shock and organ failure in the host. Most S. aureus strains carry 5 to 6 superantigen genes, including toxic shock syndrome toxin (TSST-1; or SEF); enterotoxins A to E, G to J, and R to T (SEA, etc.); and the enterotoxin-like (SEL) agents K to Q, U, V, and X (SEL-K, etc.) (36). Staphylococcal superantigen carriage versus development of clinical disease has not been extensively studied in equids; however, *tsst-1*-positive isolates have been associated with toxic shock syndrome in horses (38). In addition to toxin genes, the bacteriophage immune evasion cluster (IEC) genes (*sak*, *scn*, and *chp*) are often carried with SEA and SEL-P and inhibit host neutrophil chemotaxis, cleave host defense proteins, and inhibit host opsonization (39). Toxin and IEC gene carriage differences have not been well categorized in horse S. aureus isolates.

Biofilms are another important virulence factor in staphylococcal species. They complicate the treatment of infections and can delay healing of chronic wounds in equines (40, 41). The intercellular adhesion (*ica*) locus *icaADBC* mediates the production of polysaccharide intercellular adhesion (PIA) in staphylococcal species (42). A surface adhesion protein, BAP, has also been associated with the increased ability to form biofilms in bovine S. aureus isolates (43). Genes encoding fibronectin binding (*fnbB*) and bone sialoprotein-binding (*bbp*) proteins are additional virulence factors that influence biofilm formation. Few studies have characterized the biofilm-forming capabilities of clinical equine S. aureus isolates.

While whole-genome sequencing (WGS) has been used to retrospectively investigate and track emerging outbreaks of MRSA in human hospitals and communities (44), this technique has not been widely utilized with a large collection of MSSA and MRSA collected from horses. Few studies have examined the complete toxin gene carriage profiles of equine MRSA and MSSA strains, instead focusing on carriage of only a few toxin genes. In this study, WGS was performed on a convenience sample of 72 S. aureus isolates collected from equines that presented to the Texas A&M University Veterinary Medical Teaching Hospital over a 10-year period in order to investigate the relationship between strain type, toxin gene carriage, and antibiotic resistance.

## RESULTS

### Clinical and postmortem findings.

The most common clinical findings in the equids were subcutaneous abscesses/cellulitis or dermatitis (*n* = 17), osteomyelitis and/or joint infections (*n* = 15), pleuropneumonia (*n* = 6), and corneal ulcers (*n* = 5). The majority of the dermatitis cases were lesions located on the limbs (*n* = 10). In the 22 cases where cytologic examination of fluid or tissue aspirates was performed, neutrophilic to suppurative inflammation was observed in 19 cases, and of those, 10 cases involved coccoid bacteria. In 65% (15/23) of the cases, the death or reason for euthanasia could be attributed to bacterial infections in which S. aureus was cultured. The most common gross necropsy findings included bronchopneumonia (*n* = 6), chronic joint infections (*n* = 5), wounds or skin abscesses (*n* = 5), corneal ulcers (*n* = 2), a nasal mass (*n* = 1), and abdominal abscesses (*n* = 1). One case involved death from a severe, secondary bacterial infection at the sites of previous erythema multiforme. Histologic findings included necrosuppurative bronchopneumonia, synovitis, and skin or abdominal abscesses.

### Assembly statistics.

The average coverage achieved over the combined Illumina MiSeq runs for all isolates was 64-fold (range, 41- to 90-fold), with removal of 1 outlier of 174-fold coverage. Nine isolates had coverage estimates lower than the benchmark goal of ≥50-fold. While the overall genome coverage was high, coverage of some areas containing highly repetitive regions, such as the *spa* gene, the region flanking *egc* and *seh*, and enterotoxin gene clusters, was low in 10 isolates (09-047, 26-006, 28-062, 33-029, 36-009, 38-086, 49-063, 54-075, 58-006, and 63-019), resulting in fragmentation of genes. In these instances, either Sanger sequencing (Eton Biosciences, San Diego, CA) or conversion and BLAST query of the reads for each isolate was performed to accurately assess type and gene carriage.

### Typing characteristics.

**FIG 1:**
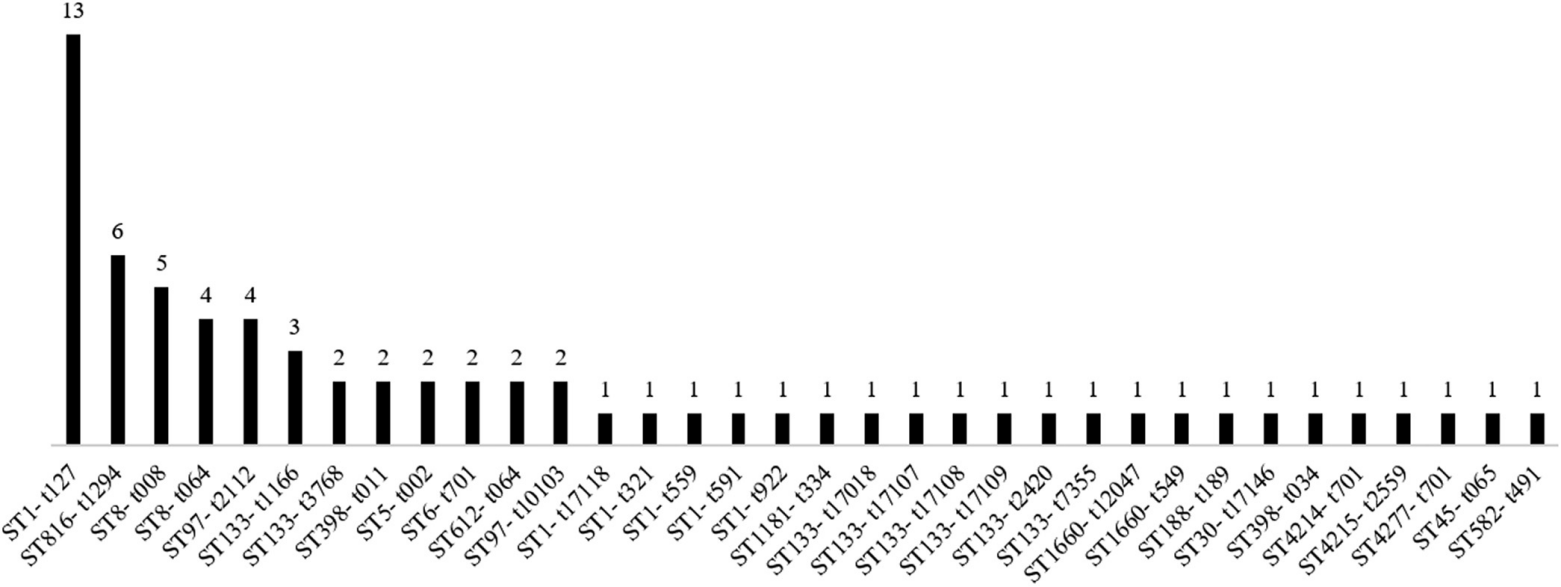
Distribution of ST and *spa* type pairings for the 72 equine S. aureus isolates.

The multilocus sequence type (MLST) and *spa* type combinations observed are illustrated in Fig. 1. The WGS *spa* types of 10 isolates did not match the Sanger sequencing, which is not uncommon in the tandem repeat regions; consequently, the Sanger *spa* types were used. Eighteen distinct ST were represented, with ST1 (*n* = 18), ST133 (*n* = 11), ST8 (*n* = 9), and ST97 (*n* = 9) most frequently encountered. Three new MLSTs were identified due to single, unique point mutations in the *aroE* (4214, 4215) and *arcC* (4277) genes. Of the 14 *mecA*-positive isolates, 64% were ST8, 14% ST612, 14% ST398, and 7% ST5. There were 2 distinct groups of ST8 MRSA, namely, 5 isolates characterized by *SCCmec* type IVa, *spa* type t008, and *dru* type dt9g; and 4 isolates having *SCCmec* IVd, *spa* t064, and *dru* dt10a. The 2 horses with travel history outside the United States cultured ST1-t127 MSSA, a major ST circulating within human communities in Canada and the northern United States and one of the predominate equine MSSA strains found in Denmark (11). New spa types included t17107, t17108, t17109, t17110, t17118, and t17146.

**FIG 2:**
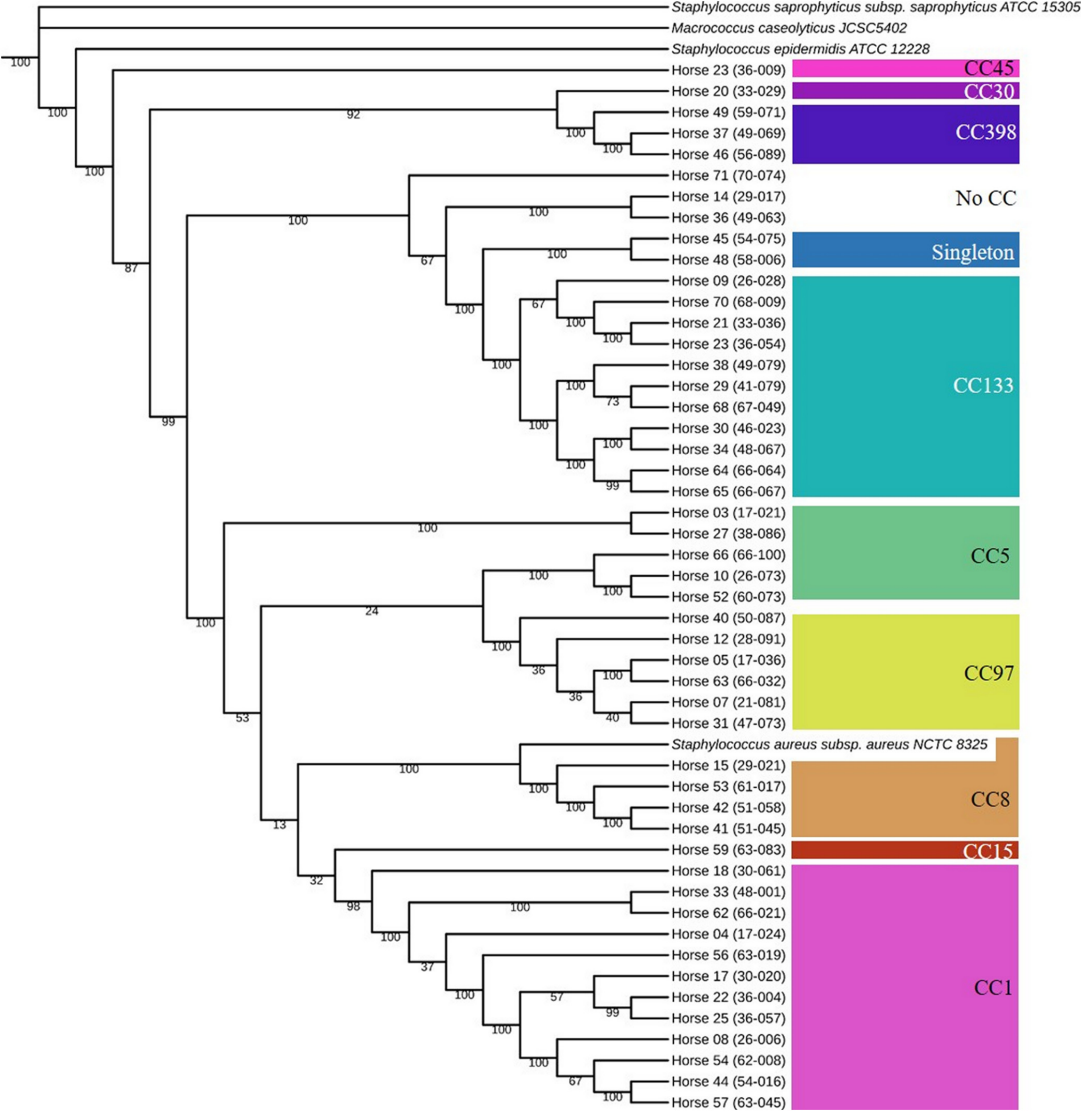
Phylogenetic tree of 50 of the equine S. aureus clinical specimens. The associated CCs are labeled at right. The numbers on the nodes are the bootstrap values. Branch lengths have been ignored.

Nine CCs were represented in this study, and groupings are visualized in the phylogenetic tree in Fig. 2, including CC1 (*n* = 19), CC133 (*n* = 12), CC8 (*n* = 12), CC97 (*n* = 9), CC5 (*n* = 5), CC398 (*n* = 3), CC15 (*n* = 1), CC45 (*n* = 1), and CC30 (*n* = 1). One ST was a singleton (ST816) and 2 STs (1660 and 4215) could not be assigned to a CC. All of the CC8 isolates were MRSA. Forty-nine rMLST types were observed, including 41 new types that were identified in this study. The most common ribosomal sequence types (rMLSTs) were 4390 (*n* = 8), 4320 (*n* = 4), 54212 (*n* = 4), 57189 (*n* = 4), and 53899 (*n* = 3).

### Biofilm production.

Eight isolates were positive for biofilm production via the crystal violet microtiter plate assay. Of the 8 positives, only 1 was MRSA and the rest were MSSA.

### Biofilm-associated gene carriage.

The intercellular adhesion genes *icaA*, *icaB*, *icaC*, and *icaD* were present in all isolates. None of the isolates carried the *bap* gene. All of the ST8, ST612, ST816, and ST1181 isolates were positive for *fnbB* (18/72, 25%). Ten isolates were positive for *bbp*. Carriage of biofilm-associated genes did not correlate with strain types or the ability to produce biofilms *in vitro* on crystal violet assay (CVA).

### Toxin and virulence gene carriage.

**FIG 3:**
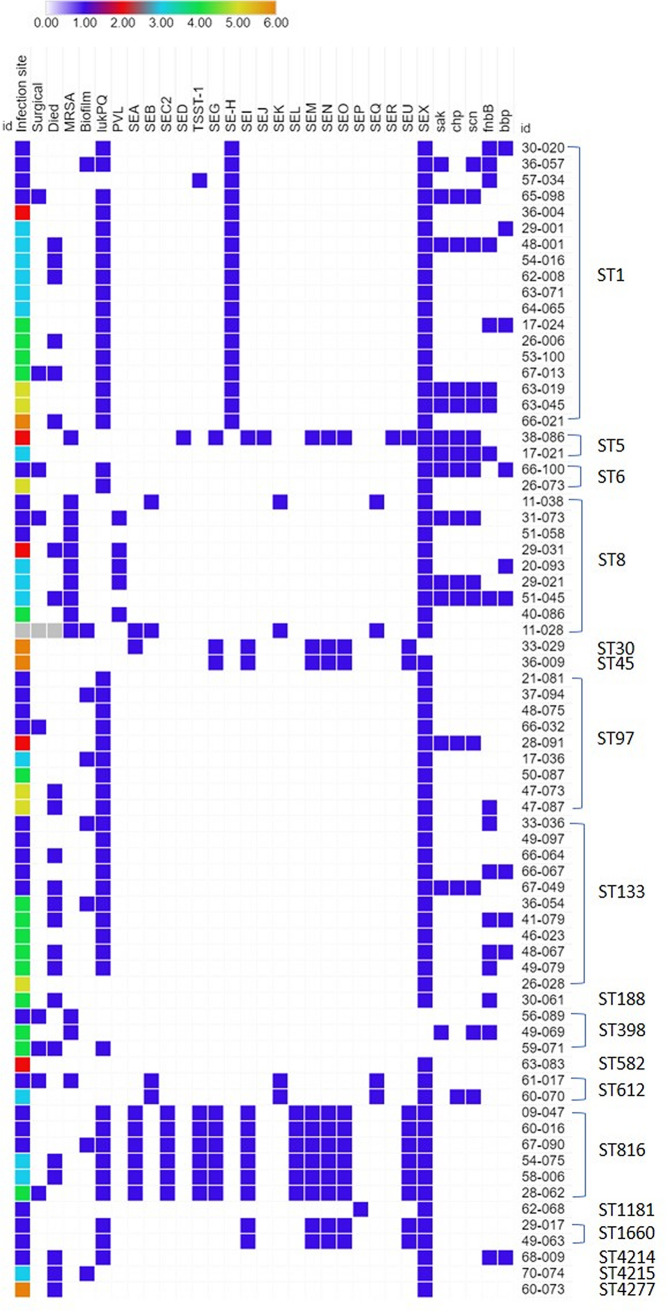
Selected toxin gene carriage for the 72 equine S. aureus isolates. For infection site category: 1 = skin, 2 = cornea, 3 = respiratory, 4 = bone/joint, 5 = blood, and 6 = other site. For other categories: 0 = false or gene not present, 1 = true or gene present. Light gray indicates the environmental isolate (11-028).

Toxin and virulence gene carriage was diverse and was correlated with typing characteristics (Fig. 3). All isolates carried aureolysin and the gamma-hemolysin A, B, and C components. Alpha-hemolysin was carried by a majority (92%, 66/72) of the isolates, as well as β-hemolysin (97%; 70/72). None of the isolates carried *eta*, *etb*, *etd*, or *lukMF’*. Panton-Valentine leucocidin was found in only five ST8-IVa-t008-dt9g isolates, which also exclusively carried the arginine catabolic mobile element (ACME). Most of the isolates carried *lukPQ* (66%; 48/72), and carriage was associated with the presence of the strain 3711 prophage (99% identity to reference LT671578.1). All of the *lukPQ*-carrying isolates were MSSA. Carriage of *lukAB* was also common (97%, 70/72), and all of these isolates also carried *lukPQ*. None of the isolates carried phenol-soluble modulin genes.

Many of the isolates carried an enterotoxin or enterotoxin-like (SEl) genes, including *sea* (*n* = 8), *seb* (*n* = 4), *sec2* variant (*n* = 6), *sed* (*n* = 1), *tsst-1* (*n* = 7), *seg* (*n* = 9), *seh* (*n* = 18), *sei* (*n* = 11), *sej* (*n* = 1), *sel-k* (*n* = 4), *sel-l* (*n* = 6), *sel-m* (*n* = 11), *sel-n* (*n* = 11), *sel-o* (*n* = 11), *sel-p* (*n* = 1), *sel-q* (*n* = 4), *ser* (*n* = 1), *sel-u* (*n* = 11), and *sel-x* (*n* = 68). The majority of isolates (96%, 69/72) carried ≥1 enterotoxin or SEL gene. The most common enterotoxin gene was *sel-x*, found in 94% of our isolates, similar to the high prevalence seen in previous equine studies (2). Enterotoxin H was found only in isolates belonging to ST1, which is again similar to previous reports (45, 46). While most isolates carried fewer than 2 toxin genes, the 6 belonging to ST816-t1294 and 1 ST5-t002 isolate carried 10 toxin genes. Several of the CC8 MRSA isolates carried *sea*, *seb*, *sel-k*, *sel-q*, and *sel-x*; the K+Q+X enterotoxin combination is one of the most commonly reported for USA300/CC8 isolates (47).

Other virulence genes included the *agr*, ACME, and IEC. The most common *agr* type was *agr* type I (57%, 41/72), followed by *agr* type III (26%, 19/72), type II (15%, 11/72), and type IV (1%; 1/72). This is similar to the prevalence seen in a study of S. aureus isolates collected from donkeys in Tunisia, where the most common *agr* types were also types I and III (45). ACME was seen in all of the ST8 isolates. The IEC genes were carried in 11 isolates, with the following ST pairings observed: A in ST30 (*n* = 1); B in ST8 (*n* = 5), ST5 (*n* = 2), ST398 (*n* = 2), ST188 (*n* = 1), and ST45 (*n* = 1); C in ST582 (*n* = 1); D in ST8 (*n* = 1); and G in ST1181 (*n* = 1). Types B and D are the most common IEC types seen in isolates of *agr* type I, while type A is commonly seen in isolates of *agr* type III (21).

### Antimicrobial resistance gene carriage.

**FIG 4:**
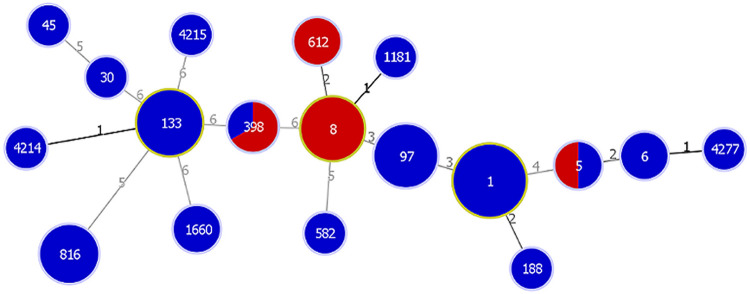
Full minimum spanning tree for the 72 equine S. aureus isolates. The red shading indicates the proportion of MRSA isolates for the indicated ST, and the blue indicates MSSA. The size of the spheres reflects the number of isolates of that type, and the numbers on the connecting lines indicate % similarity in increments of 0.5% (i.e., 1 = 99 to 99.5% similarity; 2 = 98.5% to 99% similarity).

Most of the isolates were MSSA (81%), while only 14 were MRSA. Methicillin-resistant strains were collected from two of the horses that died, but only one was infected with a strain that directly contributed to death. Of the 9 surgical site infections, 3 were attributed to MRSA strains, namely, ST8, ST398, and ST612. MSSA associated with surgical site infections included ST1 (*n* = 2), ST6, ST97, ST398, and ST816. ST97, ST398, and ST816 are associated with livestock S. aureus infections. A full minimum spanning tree based on MLST was drawn for the *mecA*-positive and -negative isolates (Fig. 4), and a summary of 14 *mecA*-positive isolates is given in Table 1. Thirteen of the methicillin-resistant isolates were phenotypically resistant to oxacillin and carried *mecA*, while one isolate (60-070) was phenotypically oxacillin susceptible but was *mecA* positive. Isolate 60-070 has a T-to-C missense mutation in the ribose-phosphate pyrophosphokinase (*prs*) gene that results in a serine-to-proline substitution at residue 292. None of the other *mecA*-positive isolates had *prs* mutations. Mutations in *prs* have been associated with phenotypic susceptibility to β-lactam drugs in *mecA*-positive, oxacillin-susceptible S. aureus (48), and 60-070 did not have mutations in other correlated loci.

**TABLE 1:** Clinical and strain typing characteristics of the 14 *mecA*-positive S. aureus isolates^*a*^

Equid ID	Isolate ID	Isolation yr	Age (yrs)	Sex	Breed	Infection site	Outcome	MLST	CC	SCC*mec* type	*spa* type	*dru* type	rMLST
Enviro	11-028	2008						8	8	IVd	t064	dt10a	53899
2	11-038	2008	14	Mare	Quarter Horse	Skin	Survived	8	8	IVd	t064	dt10a	53899
6	20-093	2010	18	Gelding	Quarter Horse	Respiratory	Survived	8	8	IVa	t008	dt9g	4320
15	29-021	2010	30	Gelding	Mixed Breed	Respiratory	Survived	8	8	IVa	t008	dt9g	4320
16	29-031	2010	4	Mare	Quarter Horse	Cornea	Died	8	8	IVa	t008	dt9g	57201
19	31-073	2011	10	Gelding	Mixed Breed	Skin^*b*^	Survived	8	8	IVa	t008	dt9g	4320
27	38-086	2012	14	Gelding	Quarter Horse	Cornea	Survived	5	5	IIa	t002	NA	4326
28	40-086	2012	6	Mare	Quarter Horse	Bone/joint	Survived	8	8	IVa	t008	dt9g	4320
37	49-069	2014	0.2	Mare	Donkey	Bone/joint	Survived	398	398	IVa	t011	dt10q	4340
41	51-045	2014	0.08	Stallion	Donkey	Respiratory	Died	8	8	IVd	t064	dt10a	53899
42	51-058	2014	6	Gelding	American Paint	Skin	Survived	8	8	IVd	t064	dt10a	54220
46	56-089	2015	16	Mare	Quarter Horse	Skin^*b*^	Survived	398	398	IVa	t011	dt10q	57692
51	60-070^*c*^	2015	31	Mare	Arabian	Respiratory	Survived	612	8	IVd	t064	dt7d	54222
53	61-017	2015	3	Gelding	Thoroughbred	Skin^*b*^	Survived	612	8	IVd	t064	dt7d	54222

a CC, clonal complex; Enviro, environmental isolate; ID, identity.

b Surgical site infection.

c Phenotypically methicillin-susceptible isolate.

Of the 39 MSSA isolates tested via commercial MIC plate assays, many were resistant to the β-lactam drugs ampicillin (34%, 13/38) and/or penicillin (39%, 14/36) due to *in vitro* production of β-lactamase. Nitrocefin testing designated 15 penicillin-susceptible MSSA isolates as resistant to β-lactams due to β-lactamase production. All phenotypic oxacillin-resistant isolates were resistant to ceftiofur, while isolate 60-070 was susceptible to ceftiofur (≤0.25 μg/ml). Two MSSA were also ceftiofur resistant and eight were intermediately resistant. For MRSA and MSSA, full or intermediate resistance to tetracycline (34%, 16/47) and gentamicin (31%, 15/49) was also prevalent. In contrast, resistance to rifampin (12%, 4/34), enrofloxacin (7/47, 15%), chloramphenicol (19%, 9/47), and doxycycline (19%, 7/36) was uncommon.

**FIG 5:**
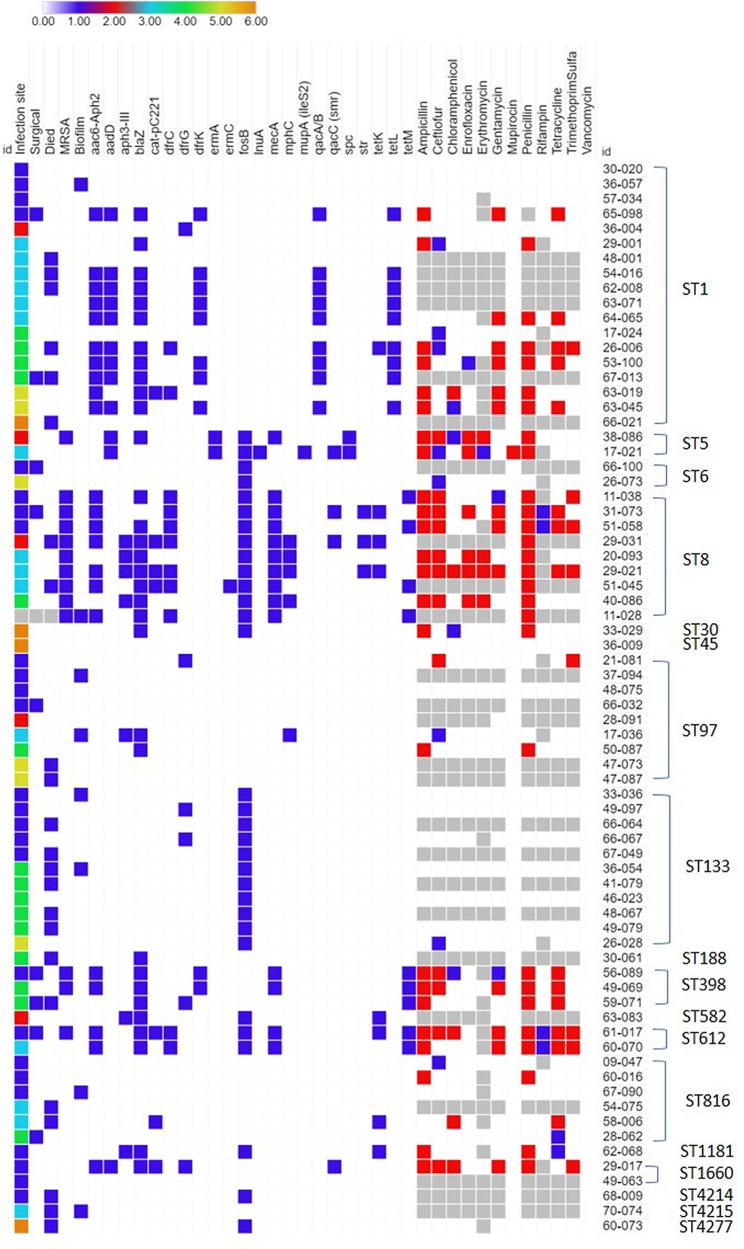
Antimicrobial resistance gene carriage profiles for the 72 equine S. aureus isolates. For infection site category: 1 = skin, 2 = cornea, 3 = respiratory, 4 = bone/joint, 5 = blood, and 6 = other site. For other categories: 0 = false or gene not present, 1 = true or gene present. Light gray indicates not applicable or not tested. For antimicrobials, white (0) is susceptible, blue (1) is intermediately resistant, and red (2) is resistant.

Antimicrobial resistance gene carriage of the isolates sequenced is summarized in Fig. 5. The β-lactamase-encoding *blaZ* gene was present in 44% (32/72) of the isolates. Of the 27 penicillin-resistant isolates identified by MIC assay and nitrocefin test, 24 carried *blaZ*. One isolate carried *blaZ* but was susceptible to penicillin. All isolates had vancomycin MICs of ≤1 μg/ml by the broth microtiter dilution method. One isolate (17-021) was resistant to mupirocin due to carriage of a *mupA* plasmid. Aminoglycoside resistance genes were carried by 40% (29/72) of the isolates. All of the isolates that showed gentamicin resistance with MIC testing carried aminoglycoside resistance genes. Seven isolates carried the chloramphenicol acetyltransferase gene encoded on the pC221 plasmid, and all of the chloramphenicol-resistant (*n* = 5) isolates determined by MIC carried this plasmid.

The tetracycline resistance genes *tetK* (*n* = 8), *tetL* (*n* = 9), and *tetM* (*n* = 9) were carried by isolates of this collection, and in 2 cases, dual carriage was also observed (*tetK + tetL* or *tetK + tetM*). The *norA*-encoded drug efflux pump was seen in all isolates. The following TMS resistance genes were commonly (38%, 27/72) observed: *dfrC* (*n* = 11), *dfrG* (*n* = 6), and *dfrK* (*n* = 10). The gene *dfrK* was found only in phenotypically TMS-susceptible isolates. Macrolide resistance genes were observed in 14% (10/72) of isolates, including *ermA* (*n* = 2), *ermC* (*n* = 1), *msrA* (*n* = 1), or dual carriage of *msrA* and *mphC* (*n* = 5). All of the erythromycin-resistant isolates identified via MIC carried resistance genes. One isolate (17-021) from a 5-year-old Paint stallion that presented with pleural effusion in 2009 carried the *lnuA* gene as well as the *mupA* (ileS2) mupirocin resistance plasmid. 17-021 was susceptible to clindamycin (MIC, ≤0.5 μg/ml) but was intermediately resistant to erythromycin (1 μg/ml).

Spectinomycin (*spc*) and streptomycin (*str*) resistance genes were rare (*n* = 2 and *n* = 3, respectively). The purported fosfomycin-resistance gene *fosB* was carried by 43% (31/72) of isolates. The 4 isolates resistant to rifampin identified by MIC all contained mutations in the *rpoB* gene associated with phenotypic resistance in S. aureus or Escherichia coli (26); 3 additional isolates not identified by MIC also carried identical mutations likely to confer rifampin resistance. Nine isolates (13%) carried *qacA/B* and 4 isolates (5%) carried *qacC*. Common carriage combinations included ST1 with aminoglycoside, β-lactamase, TMS, fluroquinolone, tetracycline, and QAC resistance genes (*n* = 9); and ST8 with aminoglycoside, β-lactamase, chloramphenicol, TMS, fluroquinolone, and tetracycline resistance genes (*n* = 2). ST133, by comparison, carried few antimicrobial resistance genes with the most common ones being *fosB* (*n* = 10) and *dfrK* (*n* = 2). A few ST97 (*n* = 7) and ST816 (*n* = 5) isolates did not carry any significant resistance genes.

## DISCUSSION

The overall prevalence of MRSA in this collection was 19%, and most of the equine MRSA cases were attributed to the USA 300 clone (ST8-IV-t008; PVL positive) (49), a clone implicated in the majority of outbreaks of community-associated MRSA in the United States, as well as the Canadian USA500 clone (ST8-IV-t064; PVL-negative). This high percentage of ST8-associated MRSA is similar to veterinary hospital cases from the northeastern United States, Ohio, and Canada attributed to USA500 as well as infections observed at French stud farms (8, 50–52). However, while previous studies with European horses found the porcine-associated ST398 to be a predominant clone (53–55), only two of the MRSA isolates in this study were ST398. The most common clonal complex in our study CC8 is one of the most frequently identified and associated with MRSA in horses (32). CC133 and CC97 groups are associated with livestock (45, 46), while CC15 has occasionally been cultured from donkeys in Tunisia (45). Strains belonging to CC5 (USA100), CC30 (USA200), and CC45 (USA600) are often associated with human infections and are the most common CC associated with blood infections and endocarditis in people (45). Additionally, CC30 is the predominant cause of mucosal infections in people, and CC45 is a common colonizer of human skin in the United States and northern Europe (45). For rMLST, several types (4390 [ST1], 4320 [ST8], 6128 [ST816], and 4340 [ST398]) were identical to the rMLST of European strains previously deposited in BIGSdb. The new rMLST types 57189 (ST816), 53899 (ST8), 54212 (ST97), 54222 (ST612), and 57200 (ST133) occurred in multiple isolates in this collection and may represent substrains that developed in the United States.

Only 11% of isolates were found to produce biofilm via the crystal violet microtiter plate assay. This result is different than what is generally seen in S. aureus isolates, although no studies have addressed biofilm production of horse clinical isolates; in human clinical isolates, overall biofilm production under *in vitro* conditions has been found to vary from 50% to 70% (56, 57). A 2009 study of 228 clinical S. aureus isolates found all strains were positive for crystal violet biofilm production, with MRSA producing stronger biofilms (58). The presence of *ica*ADBC genes is observed in the majority of isolates presented here, despite the low biofilm production. Due to the complex nature and multitude of factors mediating biofilm formation (e.g., glucose, salt, osmotic pressure, pretreatment of culture plate wells, aerobic conditions, environmental DNA (eDNA), proteins, and expression of modulating genes such as *sarA* and *rbf*), *ica*-containing species may fail to produce biofilms under testing conditions—a challenge highlighting the importance of considering both genotypic and phenotypic testing methods for biofilm formation (42, 59, 60). These discrepancies highlight the complex nature of biofilm testing and biofilm regulation (61). While carriage of biofilm genes such as *icaA* and *icaD* is high across S. aureus, clinical isolates have demonstrated variable biofilm-producing capabilities under *in vitro* testing conditions (19, 56, 58). It is possible that the *in vitro* conditions of these clinical isolates did not capture their full biofilm-forming capabilities. Additionally, biofilm production has been found to be stronger in MRSA strains than that in MSSA strains (62), and the majority of isolates in this collection were MSSA.

For toxin carriage, of interest were the *lukPQ*-positive isolates, as them confirm *lukPQ* carriage in S. aureus isolates from the United States. All isolates carrying the *lukPQ* leucocidin genes were methicillin susceptible, while the CC traditionally associated with human infections (such as CC8, CC15, and CC30 [47]) were *lukPQ* negative. None of the PVL-positive isolates also carried *lukPQ.* In the first paper describing *lukPQ* (37), the genes were found in 15% of the 87 isolates tested from the Netherlands, Italy, and Portugal; the genes were determined to be carried in ST1, ST133, ST398, and ST1660 isolates from horses. The authors did not mention the methicillin resistance status of the isolates they examined in the study; however, *lukPQ* was not seen in the subset of isolates sourced from a study by The Ohio State University (51) that consisted of 26 MRSA isolates. Consequently, most of the isolates used in the initial *lukPQ* prevalence study were likely also MRSA. A subsequent study including MSSA from healthy horses at slaughter in Spain and a WGS study of borderline oxacillin-resistant S. aureus in Germany also demonstrated a high prevalence of *lukPQ* carriage in ST1 and ST1660 isolates (63, 64). As we did not observe any *lukPQ*-positive MRSA isolates in our collection, the higher prevalence of *lukPQ* in our study of U.S. isolates is likely due to including MSSA or due to potential differences between U.S. and European MRSA isolates. Enterotoxin gene carriage was common in this collection, although none of the ST398 isolates carried toxin genes, similar to other prevalence reports in Europe that evaluated enterotoxin gene carriage (65). Carriage of *sel-x* was most common, followed by *seh+sel-x* in the ST1 isolates. All of the ST816 isolates carried a prophage that encoded *sea*, *sec2*, and *sel-l* and a novel form of *tsst-1*. ST816-t124 has been isolated from the nasal passages of healthy horses at slaughter in Spain (63).

Most of the MSSA isolates in this collection were resistant to ampicillin and penicillin. Resistance to ceftiofur (45%, 21/47), tetracycline (34%, 16/47), and gentamicin (31%, 15/49) was common. Antimicrobial resistance genes *norA*, *fosB*, *blaZ*, *aac6-aph2*, and *aadD* were common in the collection. The high level of phenotypic resistance to penicillin and tetracycline in the isolates is similar to previous reports in S. aureus from horses in Canada and Europe (8, 66). However, the percentage of gentamicin-, TMS-, and rifampin-resistant isolates in the collection is higher than the zero resistance prevalence observed in one report from Canada but lower than reports from French stud farms and a Hungarian clinic (8, 53, 66). Macrolide resistance was seen in 10 isolates. Macrolides, while contraindicated in adult animals, are primarily used for treating rhodococcal infections in foals. None of the horses that cultured macrolide-resistant isolates were being treated with a macrolide antimicrobial drug or mupirocin while in the hospital. As most isolates in this study came from adults with an unknown prior treatment history for rhodococcal infections, it is not surprising that there is some degree of macrolide resistance in this population.

Of concern is the finding of an ST5-t002 MSSA isolate with dual carriage of *lnuA* and *mupA* in an animal that had been treated only with penicillin and gentamicin. This combination of resistance genes is rare in S. aureus and to the authors’ knowledge has not been reported in an equine isolate. The profile is unusual because mupirocin and lincosamides are not routinely used in horses and suggests that there was transfer from a person to the horse. As previously mentioned, only 19% of the collection were MRSA isolates; the high association of MSSA with the majority of these equine infections, with most cultured in significant amounts and confirmed with cytologic or histopathologic findings, indicates that MSSA can be pathogenic in horses. Additionally, the diverse array of antimicrobial resistance and toxin genes in these equine MSSA isolates could contribute to patient morbidity as well as serve as reservoirs for the transfer of virulence genes between staphylococci of similar lineage.

## MATERIALS AND METHODS

### Bacterial isolates and demographics.

The collection consisted of a convenience sample of 72 S. aureus isolates collected between 2007 and 2017 from 65 horses, 6 donkeys, and 1 mule that presented to the Texas A&M University Veterinary Medical Teaching Hospital. One environmental S. aureus isolate was also included, which was collected in 2008 during a hospital procedure in a large animal ward. All patient isolates were residual diagnostic specimens with written owner consent to be retained for research purposes. Fourteen breeds of horse were represented as isolate hosts, with American Quarter horse (*n* = 29), Thoroughbred (*n* = 8), and American Paint (*n* = 7) horses being the most common. Three of the donkeys were of unspecified breed, 2 were miniature, and 1 was an American mammoth donkey. The average age of the equids was 8.4 years ± 7.6 years (range, 1 day to 31 years). Most isolates were cultured from mares (*n* = 36) and geldings (*n* = 25), with 10 collected from stallions and 1 recovered from the lung of an aborted male, full-term Clydesdale fetus. The most common culture site was the skin (*n* = 28), respiratory secretions or tissues (*n* = 15), bones or joint tissues (*n* = 15), blood (*n* = 6), and the cornea (*n* = 5). One isolate was collected from urine and 1 from lymph nodes. Two horses had documented recent travel history from Canada and France. Two horses were cultured as part of a breeding soundness exam. Nine of the cases involved infection of surgical sites, with two involving infection of joint implants and seven involving skin infections related to incisional dehiscence. Cytologic examination of fluid or tissue aspirates was performed by a clinical pathologist in 22 of the cases involving joint (*n* = 8), eye (*n* = 5), respiratory (*n* = 5), abdominal (*n* = 2), skin (*n* = 1), and urinary (*n* = 1) infections. Of the 72 equids, 23 died or were euthanized within 3 weeks of discharge from the hospital, and 21 of those received a postmortem examination.

### Antimicrobial resistance MIC testing.

The majority (68%, 49/72) of the isolates were tested via commercial veterinary MIC plate assays (Sensititre[TREK Diagnostic Systems, Inc. before 2011 or Thermo Fisher Scientific after 2011]) at the time of diagnosis against panels of commonly used antimicrobial drugs. Antimicrobial drugs tested included gentamicin, ampicillin, ceftiofur, chloramphenicol, enrofloxacin, tetracycline, trimethoprim-sulfamethoxazole, penicillin, oxacillin, amikacin, doxycycline, cefazolin, rifampin, ticarcillin, ticarcillin with clavulanic acid, ceftazidime, and erythromycin. All isolates were tested for low-level mupirocin (8 μg/ml) via 96-pin Mueller-Hinton agar dilution plate assays. Vancomycin resistance was evaluated with either Sensititre COMPGP or GPALL1F commercial broth microdilution MIC assays using the manufacturer’s instructions.

### Phenotypical characterization of biofilm production on crystal violet microtiter plate assay.

Overall biofilm production was assessed by the ability of S. aureus isolates to adhere to a 96-well microtiter plate as previously described (67). The crystal violet assay (CVA) was performed based on the referenced protocol, with the following changes: cultures were diluted 1:200 in tryptic soy broth (TSB) with 1% glucose in flat-bottomed, tissue culture-treated (TC) polystyrene microtiter plates (Falcon 96-well TC plates; Corning, USA). The wells were then carefully rinsed and left to air dry, and the dried biofilms were stained with 0.1% crystal violet dye. The optical density at 570 nm (OD_570_) values of each plate were adjusted by subtracting the average of the blank control wells that contained only TSB broth. Biofilm production cutoff values were established by the average negative-control value and two standard deviations of the negative control [Neg + 2 * (std dev)] per plate. Values below the cutoff were considered negative for biofilm production and those higher were considered positive. Microtiter plate assays were performed in technical and biological duplicate using a symmetrically inverted loading pattern to control for edge effects (68).

### DNA extraction and library preparation.

DNA was extracted from individual bacterial pellets with the DNeasy blood and tissue kit (Qiagen, Germantown, MD, USA) according to the manufacturer’s recommendations for Gram-positive bacteria, except 1 μl of a solution of 5 mg/ml lysostaphin (L7386; Sigma-Aldrich) was added to the lysis buffer per isolate. DNA was quantified via Life Technologies Qubit high sensitivity double-stranded DNA (dsDNA) assay, and all samples were normalized to 1 ng total DNA for library preparation. Sequencing libraries were prepared using the Nextera XT library prep kit (Illumina, San Diego, CA) per their standard protocol. The Qubit high-sensitivity (HS) dsDNA assay was used to determine the concentration and the Agilent TapeStation D1000 HS system was used to determine the average fragment size of the prepared libraries. All 72 samples were normalized to 4 nM, pooled for two independent runs, and sequenced on the Illumina MiSeq system with the 300 by 300 cycle v3 sequencing kit. All run data and FASTQ files were uploaded to BaseSpace (Illumina) for downstream analysis.

The online sequencing pipeline offered by the Pathosystems Resource Integration Center (PATRIC version 3.4.6; https://www.patricbrc.org) was used to assemble and annotate the S. aureus genomes (69) for analysis. Illumina MiSeq paired read libraries for each isolate were uploaded to PATRIC and *de novo* assembled using the recommended MiSeq assembly strategy parameter (69). Genome coverage of the combined MiSeq runs was estimated by comparing the total nucleotide bases per isolate to the 2.82-Mb S. aureus subsp. *aureus* NCTC 8325 reference genome in GenBank (NC_007795.1). The annotation was performed using the Rapid Annotation using Subsystem Technology tool kit (RASTtk) on the PATRIC pipeline (70).

### Strain typing and eBURST analyses.

In order to account for the propensity for alignment errors during WGS in the tandem repeat regions of the S. aureus *spa* gene, *spa* sequences were amplified via PCR as previously described (15), and the forward and reverse strands were Sanger sequenced (Eton Biosciences, San Diego, CA) to generate a consensus sequence. The tool spaTyper (http://spatyper.fortinbras.us) was used to determine the *spa* type for each isolate and compared with the sequences in the WGS assemblies; new *spa* types were submitted to the Ridom SpaServer (http://www.spaserver.ridom.de) for inclusion in the database.

Multilocus sequence typing (MLST) and ribosomal MLST (rMLST) were performed *in silico* with the assembly files as previously described (16, 71, 72), using the batch query tools on the S. aureus MLST (https://pubmlst.org/saureus) and rMLST (https://pubmlst.org/rmlst) databases as part of the BIGSdb genomics platform (17). New allele sequences were submitted for inclusion in the respective databases. Misaligned rMLST genes were Sanger sequenced for confirmation.

The MRSA isolates were screened via PCR for amplification of the *dru* segment (18), and *dru* type was assigned by querying the *mecA* cassette sequences for each isolate WGS against the online *dru* repeat and typing database (http://dru-typing.org/site/; curator, Richard V. Goering). The SCC*mec* type was assigned via BLAST query for cassettes I to VI (19). Minimum spanning trees were generated using PHYLOViZ (73), and the full goeBURST plugin was used to determine clonal complexes in the MLST database (as of March 2020).

### Toxin, antimicrobial resistance, and virulence gene queries.

The assembly files and annotated genomes were queried using the standalone BLAST+ suite version 2.6.0 and BLAST nucleotide and protein interface in the PATRIC database, respectively. The bacterial antimicrobial resistance gene database Antibiotic Resistance Gene-ANNOTation (ARG-ANNOT) and the staphylococcal VirulenceFinder 1.5 database files from the Center for Genomic Epidemiology (March 2017 versions) were used to make the database for the BLAST+ queries (74, 75). Nucleotide sequences in GenBank for *lukPQ* (LT671578.1), *qacA/B* (GU565967.1), *qacC* (M37889.1), phenol soluble modulins 1 to 4 (BK006301.1), and *chp* (AF285146.1) were also added to the database. A positive hit for a gene was defined as a query having at least 95% identity with and covering at least 90% of the length of the database gene reference. The *agr* and IEC types were assigned based on a BLAST query using primer sequences from the previously described PCR typing methods (39, 76). The UniProt protein sequences for the *ica* genes (Q9RQP9, Q9RQP7, Q9RQP6, and Q9RQP8), *fnbB* (A0A0H2XKG3), *bap* (Q79LN3), and *bbp* (Q14U76) were queried using the PATRIC BLAST feature. Due to nucleotide variation, a positive hit for a protein was defined as a query having at least 95% identity with and covering at least 90% of the length of the UniProt reference. To ensure complete coverage of genes in repetitive areas, the raw MiSeq FASTQ files were converted to FASTA files with fastq2fasta (TM Software, Inc., Arcadia, CA) and requeried as above; a cutoff of >90% identity to a known toxin or virulence proteins cataloged in Uniprot and GenBank was used to identify the genes. Potential regions containing prophages were analyzed with PHAST (77). Toxin and antimicrobial resistance gene carriage heatmaps were made using Morpheus (https://software.broadinstitute.org/morpheus).

10.1128/mSphere.00196-20.1 DATA SET S1 NCBI accession numbers, clinical demographics, antimicrobial drug testing, biofilm, and gene carriage characteristics for the 72 S. aureus isolates. Download Data Set S1, XLSX file, 0.1 MB. Copyright © 2021 Little et al. 2021 Little et al. https://creativecommons.org/licenses/by/4.0/ This content is distributed under the terms of the Creative Commons Attribution 4.0 International license.

### Data availability.

All assemblies are freely available in the public workspace of PATRIC under the name “Equine S. aureus isolates” and in the NCBI GenBank database under BioProject PRJNA604020 and accessions JAAFKO000000000 to JAAFNH000000000. Illumina reads were deposited under accessions SRR14923786 to SRR14923857.
